# Supplementary material for: Activation energy and force fields during topological transitions of fluid lipid vesicles
Source: Commun Phys. 2022 Nov 12;5(1):283. doi: 10.1038/s42005-022-01055-2 (PMC9660165; doi:10.1038/s42005-022-01055-2)
Supplement: Supplementary file 2 — Supplementary Information [file 42005_2022_1055_MOESM2_ESM.pdf]

## SUPPLEMENTARY INFORMATION

# Activation energy and force fields during topological transitions of fluid lipid vesicles

Matteo Bottacchiari, Mirko Gallo, Marco Bussoletti and Carlo Massimo Casciola*

*Department of Mechanical and Aerospace Engineering, Sapienza Università di Roma, Rome, Italy*

* carlomassimo.casciola@uniroma1.it

## SUPPLEMENTARY DISCUSSION

### Minimal energy pathway with unconstrained volume

In the main text, the MEP for the topological transition between two spherical vesicles and a dumbbell has been obtained constraining the volume to a prescribed value, which is, e.g., physically determined by the osmotic conditions. This restriction, coupled with the conservation of the total surface area $A$, resulted in a MEP with a fixed reduced volume $v = V/(\pi\, D_{\rm ve}^3/6)$. Since the two initial spheres had the same diameter $D$, the total $D_{\rm ve} = \sqrt{A/\pi}$ was actually $\sqrt{2}D$, which together with the total volume $V$, resulted in $v = 1/\sqrt{2}$. Clearly, the constraints on membrane volume and area play a significant role in determining the MEP, given that the initial and final equilibrium shapes are determined by the reduced volume. In principle, the generality of our approach allows the exploration of different MEPs for any given $v$. However, there are cases where the total volume is not conserved. We show here the MEP in absence of such a constraint, allowing the reduced volume to vary along the pathway. In this case, the initial configuration is the same as the main text, whereas the final one approaches a single sphere with a surface area that is the sum of those of the two disjointed initial spheres. The fusion/fission mechanism appears to be the same, Supplementary Fig. S1: the forward barrier is mainly topological and is associated with the formation of a catenoid-like neck, whereas the backward one builds up continuously with the membrane deformation. Supplementary Fig. S2 shows (minus) the elastic reaction force of the membrane along selected membrane configurations.

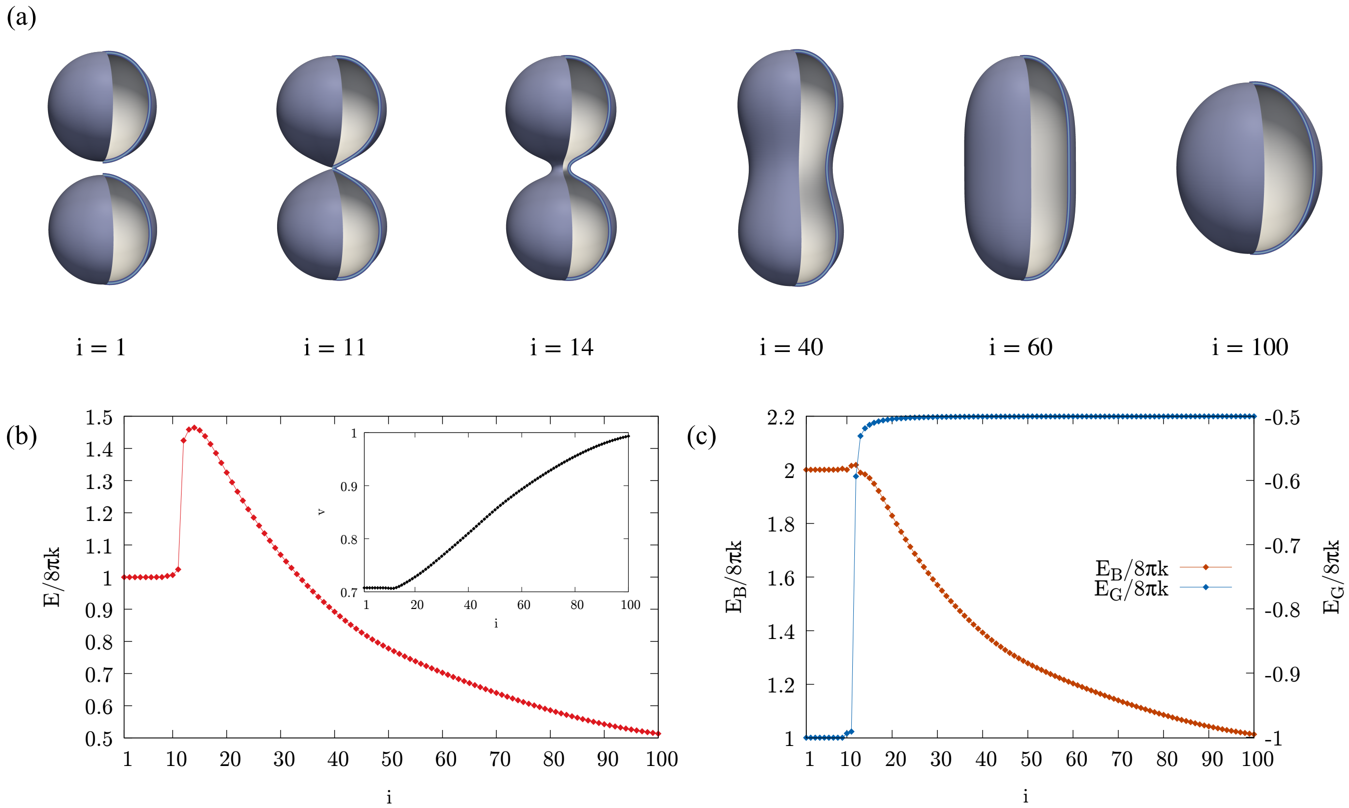


**Supplementary Fig. S1 The minimal free energy path with no volume constraint.** The pathway connects two spheres of radius $R^* = 87.5$ with a spherical vesicle of radius $\sqrt{2}R^*$, $k = -k_{\rm G}$, $m^* = 0$. In this case, the volume constraint has been relaxed like in the case, e.g., where the vesicle is made of a completely permeable membrane. This axially symmetric result is obtained with the string method using a $[0,\ 140] \times [-240,\ 240]$ computational domain in the $r^* - z^*$ plane with a grid of $210 \times 720$ nodes per image, $N = 100$ images and $1/\lambda \approx 247.5$. **a** Six vesicle shapes along the minimal energy path, identified by their image number $i = (N-1)\alpha_i + 1$, being $\alpha$ the string parameter (equal arc-length parameterization). **b** The free energy, equation (S.1), along the path. The saddle point consists of two spherical vesicles connected by a neck. In the inset: the reduced volume along the string. **c** Bending and Gaussian energy contributions along the path.

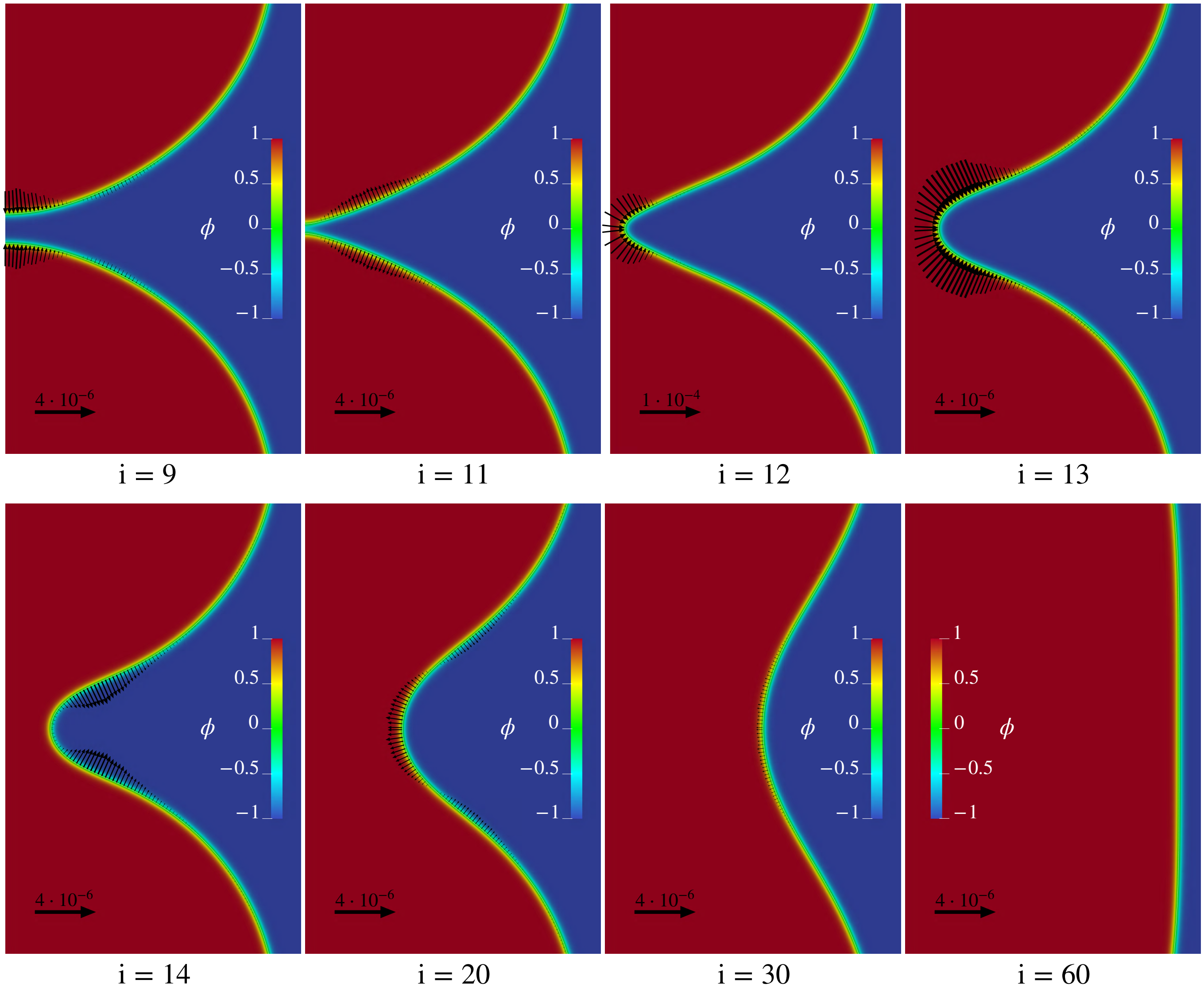


**Supplementary Fig. S2 Force fields along the MEP with no volume constraint.** Detailed views in the $r^* - z^*$ plane of the vesicle configurations. The index $i = (N-1)\alpha_i + 1$ numbers the images on the string of Supplementary Fig. S1. Vectors, which are plotted for clarity only on the $\phi = 0$ isoline, provide the force field $\mathbf{f}^*$ required to keep the vesicle in equilibrium in the given configuration, balancing the internal elastic reaction. The contours depict the field $\phi$. For better visibility, vectors are scaled according to the reference arrow in each plot.

### Fission induced by a non-zero spontaneous curvature

The fusion and fission barriers illustrated in the main text require the action of some external agent to be overcome. These agents are typically protein systems whose mode of operation may differ considerably from case to case, e.g. by involving active motors or simply modifying the membrane's spontaneous curvature. Recently, controlled fission of cell-sized vesicles by membrane-bound proteins at low density has been reported in Ref. [1]. These proteins induce a non-zero spontaneous curvature $m$ which can lead to the spontaneous fission of the vesicle. Also in our case, the introduction of a sufficiently high $m$ leads to the same behavior, lowering the fission barrier. Using a prolate shape taken from the fission branch of the MEP of the main text, Fig. 2, as the initial condition for the Allen-Cahn dynamics, equation (20), and adding a spontaneous curvature $m^* = 0.02$, the vesicle undergoes fission, Supplementary Fig. S3. The fact that fission takes place spontaneously along the relaxation process demonstrates that no residual energy barrier separates the prolate shape and the final two-sphere configurations. In general, increasing $m$ leads to a reduction of the fission barrier, and, above a critical threshold, the process becomes spontaneous.

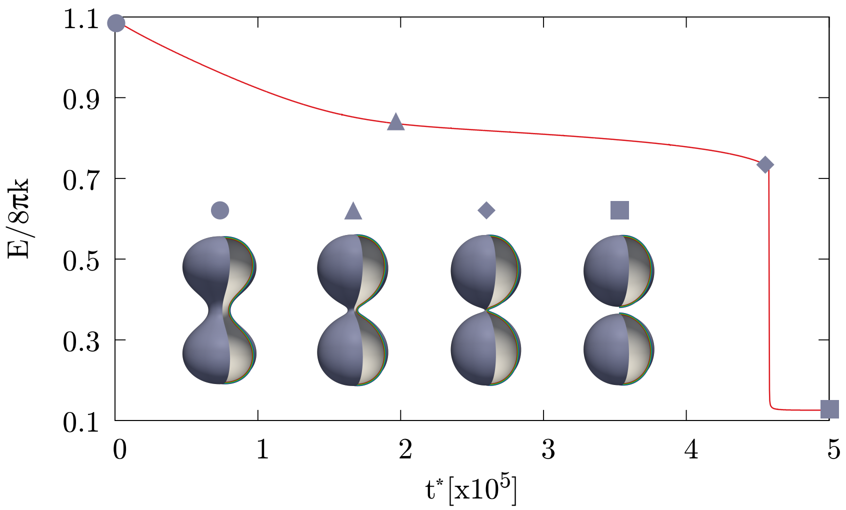


**Supplementary Fig. S3 Spontaneous curvature-induced fission.** The prolate divides into two, identical spheres with $m^* = 0.02$. The final sphere radius is $R^* = 87.5$. Evolution obtained by the Allen-Cahn dynamics including the Gaussian energy term. For this simulation we used a $[0, 96] \times [-245, 245]$ axisymmetric $r^* - z^*$ domain with a grid of $192 \times 980$ nodes, $\epsilon^* = 1.5h^* = 1$, $1/\lambda = 247.5$, $M^* = 100$ and time step $dt^* = 0.025$.

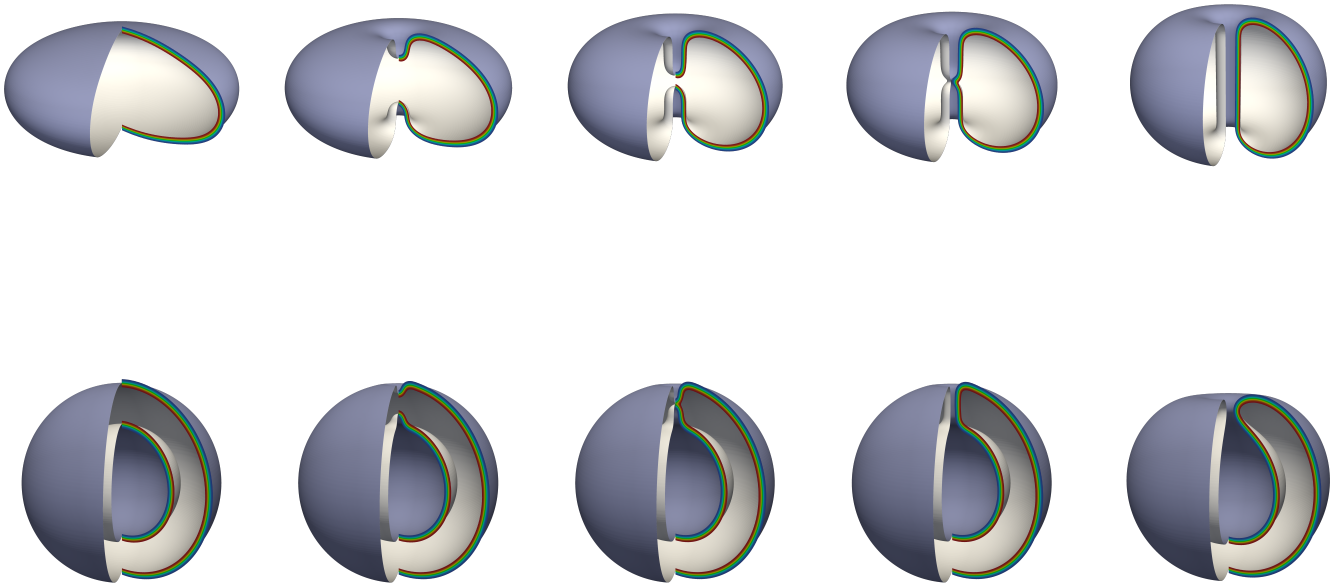

**Supplementary Fig. S4 Forced topology change.** Top: Successive configurations along the topology change from an oblate vesicle into a torus (reduced volume $v = 0.84$). Bottom: Successive configurations along the topology change from two nested, concentric spheres to a stomatocyte (the ratio of the sphere radii is $R_{in}/R_{ex} = 0.6$ and the stomatocyte reduced volume is $v = 0.49$).

### Different systems and external forces

In order to demonstrate the generality of the topological transformations that can be treated with the approach described in the main text, we address here the transformation of an oblate vesicle into a toroid and two nested spheres fusing into a stomatocyte, Supplementary Fig. S4. In these cases, the topology change is driven by an external force $\mathbf{f}$ acting as forcing to the Allen-Cahn dynamics, equation (20), derived from a suitable energy $E_{ex}[\phi]$ and exploiting the expression derived in Section Methods of the main text, namely $\mathbf{f}_{ex} = -\delta E_{ex}/\delta\phi \nabla\phi$.

## SUPPLEMENTARY METHODS

### Additional details on the sharp interface limit

The phase-field $\phi(\mathbf{x})$ is a regular function, defined on a domain $\Omega \in \mathbb{R}^3$, which takes values in the range $(-1, 1)$. The energy given by the functional $E[\phi]$ is attached to each field configuration, as specified by the equations

$$E[\phi] = E_{\rm B}[\phi] + E_{\rm G}[\phi], \tag{S.1}$$

with

$$E_{\rm B}[\phi] = k\,\frac{3}{4\sqrt{2}}\,\epsilon \int_\Omega \Psi_{\rm B}^2\, dV, \tag{S.2}$$

$$\Psi_{\rm B} = \nabla^2\phi - \frac{1}{\epsilon^2}(\phi^2 - 1)(\phi + \sqrt{2}\epsilon m) \tag{S.3}$$

and

$$E_{\rm G}[\phi] = k_{\rm G}\,\frac{35}{16\sqrt{2}}\,\epsilon^3 \int_\Omega \Psi_{\rm G}\, dV, \tag{S.4}$$

$$\Psi_{\rm G} = \frac{\boldsymbol{\nabla}|\boldsymbol{\nabla}\phi|^2 \cdot \boldsymbol{\nabla}|\boldsymbol{\nabla}\phi|^2}{2} - (\boldsymbol{\nabla}|\boldsymbol{\nabla}\phi|^2 \cdot \boldsymbol{\nabla}\phi)\nabla^2\phi \; + \; |\boldsymbol{\nabla}\phi|^2\left[(\nabla^2\phi)^2 + \boldsymbol{\nabla}\phi\cdot\boldsymbol{\nabla}\nabla^2\phi - \frac{\nabla^2|\boldsymbol{\nabla}\phi|^2}{2}\right]. \tag{S.5}$$

In the limit of vanishing $\epsilon$ the field energy will be shown below to converge to the sharp interface energy of the Canham-Helfrich model, see equation. (1) in the main text, for a membrane identified with the mid surface implicitly defined by the field isosurface $\phi(\mathbf{x}) = 0$. In this case, the various parameters entering the expression for the energy will be interpreted as follows: $m$ is the spontaneous curvature of the membrane, assumed to be closed, taken to be positive if the membrane bulges towards the exterior; $k$ is the bending rigidity; $k_{\rm G}$ is the Gaussian curvature modulus (for definiteness, $k_{\rm G} = -k$ will be assumed, as suggested by the experimental data, see the main text for details). Hence the equilibrium configurations of a Canham-Helfrich membrane can be obtained either by minimizing the Canham-Helfrich functional with respect to the membrane configuration or, alternatively, by minimizing the phase-field energy functional in the limit of vanishing $\epsilon$. As explained in the main text, apart from practical advantages in the minimization process, the phase-field approach presents the main advantage over the sharp interface description of allowing for the membrane topological transitions.

More technically, it is shown below that an ansatz for the phase function of the form

$$\phi(\mathbf{x}) = f\left(\frac{d(\mathbf{x})}{\epsilon}\right), \tag{S.6}$$

where $d(\cdot)$ is the signed distance from the (assigned) membrane mid-surface configuration $\Gamma = \{\mathbf{x} : \phi(\mathbf{x}) = 0\}$ and $f$ is such that $f(\pm\infty) = \pm 1$ and $f(0) = 0$: i) does indeed allow to find a minimizer of the field energy for small enough $\epsilon$ and given mid-surface geometry; ii) leads to a limiting, sharp interface form of the field energy which coincides with the Canham-Helfrich energy, which is a functional of the membrane configuration. From the discussion, it will become clear that the parameter $\epsilon > 0$ is related to the width of the diffuse interface and, thus, to the small, but finite, membrane thickness.

We choose to define the signed distance such that $\mathbf{n} = \boldsymbol{\nabla} d$ computed on $\Gamma$ is the inward-pointing unit normal to the vesicle. It is worth noticing that the signed distance function satisfies the eikonal equation, namely $|\mathbf{n}| = 1$. As anticipated, setting $d^*(\mathbf{x}) = d(\mathbf{x})/\epsilon$, we also require that $\lim_{d^*\to\pm\infty}\phi = \pm 1$ and $\phi = 0$ for $d = 0$. Therefore, $\pm 1$ are the values for the stable phases of the inside and outside bulk and the level set $\phi = 0$ identifies the membrane mid-surface.

After introducing the membrane characteristic length $D_{\rm ve} = \sqrt{A/\pi}$, where $A$ is the membrane area, the sharp interface limit corresponds to $\lambda = \epsilon/D_{\rm ve} \to 0$. Since $\boldsymbol{\nabla}\phi(\mathbf{x}) = f'(d^*(\mathbf{x}))\,\mathbf{n}/\epsilon$, where the prime denotes the derivative with respect to $d^*(\mathbf{x})$, a direct computation leads to

$$E_{\rm B}[\phi] = \; k\,\frac{3}{4\sqrt{2}}\,\lambda\int_{\bar\Omega}\left[\frac{1}{\lambda^2}\Big(f'' - (f^2-1)f\Big) \;+\; \frac{1}{\lambda}\Big(f'\,\bar{\boldsymbol{\nabla}}\cdot\mathbf{n} + (1-f^2)\sqrt{2}\bar m\Big)\right]^2 d\bar V, \tag{S.7}$$

$$E_{\rm G}[\phi] = k_{\rm G}\,\frac{35}{16\sqrt{2}}\int_{\bar\Omega}\frac{f'^4}{\lambda}\left[(\bar{\boldsymbol{\nabla}}\cdot\mathbf{n})^2 + \mathbf{n}\cdot\bar{\boldsymbol{\nabla}}(\bar{\boldsymbol{\nabla}}\cdot\mathbf{n})\right]d\bar V, \tag{S.8}$$

where the bar indicates lengths normalized with $D_{\rm ve}$. Following [2], in order to minimize $E = E_{\rm B} + E_{\rm G}$ as $\lambda \to 0$ the phase-field function is expanded as

$$\phi(\mathbf{x}) = f(d^*(\mathbf{x})) = f_0(d^*(\mathbf{x})) + \lambda f_1(d^*(\mathbf{x})) + R_\lambda(d^*(\mathbf{x})) \tag{S.9}$$

where $R_\lambda(d^*(\mathbf{x})) = O(\lambda^2)$, obtaining

$$\begin{aligned} E_{\rm B}[\phi] = \; & k\,\frac{3}{4\sqrt{2}}\,\lambda\int_{\bar\Omega}\left[\frac{1}{\lambda^2}\Big(f_0'' - (f_0^2-1)f_0\Big) + \right. \\ & \left. + \;\frac{1}{\lambda}\Big(f_1'' - f_1(3f_0^2-1) + f_0'\,\bar{\boldsymbol{\nabla}}\cdot\mathbf{n} + (1-f_0^2)\sqrt{2}\bar m\Big) + h\right]^2 d\bar V\;, \end{aligned} \tag{S.10}$$

$$E_{\rm G}[\phi] = k_{\rm G}\,\frac{35}{16\sqrt{2}}\int_{\bar\Omega}\left(\frac{f_0'^4}{\lambda} + 4f_0'^3 f_1' + \lambda g\right)\left[(\bar{\boldsymbol{\nabla}}\cdot\mathbf{n})^2 + \mathbf{n}\cdot\bar{\boldsymbol{\nabla}}(\bar{\boldsymbol{\nabla}}\cdot\mathbf{n})\right]d\bar V\;, \tag{S.11}$$

with $g = g(d^*(\bar{\mathbf{x}}))$ and $h$ a combination of functions factorized in the form $\eta(d^*(\bar{\mathbf{x}}))\xi(\bar{\mathbf{x}})$. Therefore, both $g$ and $h$ are $O(1)$ in $\lambda$. Expansion (S.9) entails the ordering of the two partial energy functionals as power series in the small parameter $\lambda$. Therefore, in order to minimize the otherwise dominating bending energy (order $1/\lambda^3$), the leading order term $f_0$ must satisfy

$$f_0'' = (f_0^2 - 1)f_0, \tag{S.12}$$

i.e.,

$$f_0(d^*(\mathbf{x})) = \tanh\left(\frac{d(\mathbf{x})}{\epsilon\sqrt{2}}\right), \tag{S.13}$$

which identifies $\epsilon$ as directly proportional to the interface width. Note that, once $f_0$ is determined, the order $\lambda^{-1}$ contribution to the Gaussian energy is fixed. Concerning the bending energy, we are left with

$$E_{\rm B}[\phi] = \; k\,\frac{3}{4\sqrt{2}}\,\lambda\int_{\bar\Omega}\left[\frac{1}{\lambda}\Big(f_1'' - f_1(3f_0^2-1) + f_0'\,\bar{\boldsymbol{\nabla}}\cdot\mathbf{n} + (1-f_0^2)\sqrt{2}\bar m\Big) + h\right]^2 d\bar V \;=$$

$$k\frac{3}{4\sqrt{2}}\frac{1}{\lambda}\int_{\bar\Omega}\left[\left[f_1'' - f_1(3f_0^2-1)\right]^2 + 2f_0'\left[f_1'' - f_1(3f_0^2-1)\right]\left[\bar{\boldsymbol{\nabla}}\cdot\mathbf{n} + 2\bar m\right] + {f_0'}^2\left[\bar{\boldsymbol{\nabla}}\cdot\mathbf{n} + 2\bar m\right]^2 + \lambda\tilde h\right]dV, \tag{S.14}$$

where we have used

$$\sqrt{2}f_0' = (1 - f_0^2)\;, \tag{S.15}$$

and $\tilde h = O(1)$ shares the same structure of $h$.

Now, considering that

$$f_0''' = f_0'(3{f_0}^2 - 1)\ , \tag{S.16}$$

one finds that

$$\int_{\bar\Omega} 2f_0'\big[f_1'' - f_1(3f_0^2-1)\big]\big[\bar{\boldsymbol\nabla}\cdot\mathbf{n} + 2\bar m\big]\,d\bar V \;=\; \int_{\bar\Omega} 2\big[f_0'f_1'' - f_0'''f_1\big]\big[\bar{\boldsymbol\nabla}\cdot\mathbf{n} + 2\bar m\big]\,d\bar V. \tag{S.17}$$

Integration by parts leads to

$$\begin{aligned}
&2\int_{\bar\Omega}\big[f_1f_0'''\big]\big[\bar{\boldsymbol\nabla}\cdot\mathbf{n}+2\bar m\big]\,d\bar V = \;2\lambda\int_{\bar\Omega}\big[f_1\bar{\boldsymbol\nabla}f_0''\cdot\mathbf{n}\big]\big[\bar{\boldsymbol\nabla}\cdot\mathbf{n}+2\bar m\big]\,d\bar V = \\
&=\;-2\int_{\bar\Omega}\big[f_1'f_0''\big]\big[\bar{\boldsymbol\nabla}\cdot\mathbf{n}+2\bar m\big]\,d\bar V\;-\;2\lambda\int_{\bar\Omega}\big[f_1f_0''\big]\bar{\boldsymbol\nabla}\cdot\big[\mathbf{n}\big(\bar{\boldsymbol\nabla}\cdot\mathbf{n}+2\bar m\big)\big]\,d\bar V = \\
&=\;2\int_{\bar\Omega}\big[f_1''f_0'\big]\big[\bar{\boldsymbol\nabla}\cdot\mathbf{n}+2\bar m\big]\,d\bar V\;+\;2\lambda\int_{\bar\Omega}\big[f_1'f_0'-f_1f_0''\big]\bar{\boldsymbol\nabla}\cdot\big[\mathbf{n}\big(\bar{\boldsymbol\nabla}\cdot\mathbf{n}+2\bar m\big)\big]\,d\bar V
\end{aligned}$$

and therefore

$$\int_{\bar\Omega} 2f_0'\big[f_1'' - f_1(3f_0^2-1)\big]\big[\bar{\boldsymbol\nabla}\cdot\mathbf{n}+2\bar m\big]\,d\bar V \;=-\;\int_{\bar\Omega} 2\lambda\big[f_1'f_0' - f_1f_0''\big]\bar{\boldsymbol\nabla}\cdot\big[\mathbf{n}\big(\bar{\boldsymbol\nabla}\cdot\mathbf{n}+2\bar m\big)\big]\,d\bar V.$$

Since the contribution of the above integral is of higher order, substitution in (S.14) shows that minimizing the $\lambda^{-1}$-order bending energy term is tantamount to solving the equation

$$f_1'' - (3f_0^2-1)f_1 = 0. \tag{S.18}$$

Two independent solutions are easily found. Given Eq. (S.16), $f_{1a} = f_0'$ is clearly one of them, while the other can be sought in the form $f_{1b}(d^*) = w(d^*)f_0'(d^*)$. Thus, equation (S.18) implies

$$f_0'w'' + 2f_0''w' = 0\,, \tag{S.19}$$

which, after setting $w' = z$ and still considering Eqs. (S.12) and (S.15), is reduced to

$$z' = 2\sqrt{2}f_0\,z\ . \tag{S.20}$$

The solution is $z(d^*) = \cosh^4\left(\dfrac{d^*}{\sqrt{2}}\right)$, i.e.

$$w(d^*) = \frac{1}{32}\big[12d^* + 8\sqrt{2}\sinh(\sqrt{2}\,d^*)) + \sqrt{2}\sinh(2\sqrt{2}\,d^*)\big]\ , \tag{S.21}$$

and therefore

$$f_{1b}(d^*) = \frac{1}{32\sqrt{2}}\left[12d^* + 8\sqrt{2}\sinh(\sqrt{2}\,d^*)) + \sqrt{2}\sinh(2\sqrt{2}\,d^*)\right]\operatorname{sech}^2\left(\frac{d^*}{\sqrt{2}}\right) \tag{S.22}$$

($f_0'(d^*) = \operatorname{sech}^2(d^*/\sqrt{2})/\sqrt{2}$), where, due to the factors $\sqrt{2}$ in the arguments, $\lim_{d^*\to\pm\infty} f_{1b}(d^*) = \pm\infty$. The two constants in the general solution of (S.18),

$$f_1 = Af_{1a} + Bf_{1b}, \tag{S.23}$$

are determined as follows. Since $f_0(\pm\infty) = \pm1$, $B = 0$ by requiring that $\lim_{d^*\to\pm\infty}\phi = \pm1$, and, given that $f_0(0) = 0$, $A = 0$ by requiring that $\phi = 0$ on the membrane mid-surface. Therefore, $f_1 \equiv 0$, and we are left with

$$E_{\rm B}[\phi] = k\frac{3}{4\sqrt{2}}\int_{\bar\Omega}\frac{f_0'^2}{\lambda}(\bar{\boldsymbol\nabla}\cdot\mathbf{n}+2\bar m)^2\,d\bar V\;+\;O(\lambda), \tag{S.24}$$

$$E_{\rm G}[\phi] = k_{\rm G}\,\frac{35}{16\sqrt{2}}\int_{\bar\Omega}\frac{f_0'^4}{\lambda}\left[(\bar{\boldsymbol\nabla}\cdot\mathbf{n})^2 + \mathbf{n}\cdot\bar{\boldsymbol\nabla}(\bar{\boldsymbol\nabla}\cdot\mathbf{n})\right]d\bar V\;+\;O(\lambda^2), \tag{S.25}$$

where the terms $O(\lambda)$ and $O(\lambda^2)$ in Eqs. (S.24) and (S.25), respectively, follow from the property $\int_{\bar{\Omega}} \eta(d^*(\bar{x}))\xi(\bar{x})\, d\bar{V} = O(\lambda)$, for each $\eta(d^*(\bar{x})) = O(1)$ such that $\eta(\pm\infty) = 0$ sufficiently fast. Denoting with $k_1$ and $k_2$ the principal curvatures of the membrane, we have $\boldsymbol{\nabla} \cdot \mathbf{n} = -(k_1 + k_2) = -2M$ and $\mathbf{n} \cdot \boldsymbol{\nabla} k_i = k_i^2$, with the result that $(\boldsymbol{\nabla} \cdot \mathbf{n})^2 + \mathbf{n} \cdot \boldsymbol{\nabla}(\boldsymbol{\nabla} \cdot \mathbf{n}) = 2k_1k_2 = 2G$. Now, after noticing that for $\lambda \to 0$

$$\frac{f_0'^2(\bar{d}(\mathbf{x})/\lambda)}{\lambda} \xrightarrow{\mathcal{W}} \frac{2\sqrt{2}}{3}\, \delta(\bar{d}(\mathbf{x})), \tag{S.26}$$

$$\frac{f_0'^4(\bar{d}(\mathbf{x})/\lambda)}{\lambda} \xrightarrow{\mathcal{W}} \frac{8\sqrt{2}}{35}\, \delta(\bar{d}(\mathbf{x})), \tag{S.27}$$

where the limits should be understood in a weak sense and $\delta(x)$ is the Dirac delta function, we are left with

$$\lim_{\lambda \to 0} E[\phi] \;=\; 2k \int_\Gamma (M - m)^2\, dS \;+\; k_{\mathrm{G}} \int_\Gamma G\, dS\,, \tag{S.28}$$

which indeed coincides with the Canham-Helfrich functional given as Eq. (1) in the main text.

From the above analysis, denoting $\phi_e(\mathbf{x}, \lambda) = \operatorname{argmin} E[\phi, \lambda]$, it follows that the surface

$$\Gamma_e = \left\{ \mathbf{x} : \lim_{\lambda \to 0} \phi_e(\mathbf{x}, \lambda) = 0 \right\}$$

is a minimizer of the sharp interface energy whenever $\Gamma_e$ is sufficiently smooth to allow the evaluation of the Canham-Helfrich functional.

The above reasoning can be extended to the case where the field is subject to the constraint on the vesicle areas and enclosed volumes, with the phase-field area and volume estimators given in Eqs. (19) and (20) in the main text.

Although in Section Results and Discussion of the main text we have matched the lipid bilayer thickness with the diffuse interface width, $\ell_{\mathrm{pf}} = 6\epsilon = \ell_{\mathrm{me}} = 5\,\mathrm{nm}$, no direct physical interpretation of the phase-field $\phi$ has been given. However, in [55] the authors suggest the field to be a normalized lipid density. In any case, regardless of this physical interpretation, we like to point out that this approach can also be used to separately describe the two constituent monolayers, each with its own elastic energy, and that the full-scale evolution of a large membrane as described in this paper could be used as a base building block to realize multiscale simulations that can locally take into account the atomistic dynamics in the the limited region where lipid rearrangement takes place.

Lastly, it is worth noticing that it is possible to consider other phase-field functionals able to asymptotically reproduce the Canham-Helfrich Gaussian energy in the sharp-interface limit ($\lambda << 1$). However, many of these formulations only work as post-processors, in the sense that they can monitor the Gaussian energy on a given solution but are inappropriate for coupling with the evolution. Indeed, the Gaussian energy must be of the proper order in $\lambda$ in order not to spoil the dominating contributions, as follows from the above asymptotic analysis. Moreover, the functional and the relative functional derivative should be smooth. Indeed, since $\mathbf{n} = \boldsymbol{\nabla}\phi/|\boldsymbol{\nabla}\phi|$, one may be tempted to obtain a phase-field Gaussian energy which involves division by $|\boldsymbol{\nabla}\phi|$. This is likely to introduce some singularity in the energy density, which in turn may lead to spurious peaks in the Gaussian energy during the topological transitions. Such singularities may also be inherited by the functional derivative, with issues in computing the evolution. Finally, phase-field Gaussian energy density based on the assumption that $\phi(x) = \tanh(d(x)/\epsilon\sqrt{2})$ may lead to cancellation errors, even if used just as post-processors, as explained in [2]. This once again points to the importance of a formulation based on the weaker assumption (S.6).

## Numerical validation

In literature [3], it is well known that, in the presence of the bending energy alone, two initially close-by spheres merge together during the Allen-Cahn dynamics, equation (20). If the area and volume constraints are included, at the steady state, a dumbbell shape with a reduced volume $v = 1/\sqrt{2}$ is obtained. This happens because the bending energy of the two spheres is greater than that of the obtained dumbbell shape. Moreover, such a numerical experiment shows that there is no energy barrier for the process. This behavior is no longer possible if the Gaussian energy is also included. Indeed, in this case, the whole Canham-Helfrich energy of two spheres is less than that of the dumbbell shape. Therefore, our first numerical validation experiment is to repeat this simulation including the new Gaussian energy term, equation (S.4). As shown in Supplementary Fig. S5, two spheres of equal radius $R^* = 10$ at distance $R^*/2$ from each other do not merge. This simulation has been carried out in a $[0, 40] \times [0, 40] \times [0, 66]$ full 3D $x^* - y^* - z^*$

domain with a grid of $40 \times 40 \times 66$ nodes (grid length interval $h^* = 1$), $\epsilon^* = h^* = 1$, $1/\lambda = 20\sqrt{2}$, $M^* = 1$ and time step $dt^* = 0.8$. In Supplementary Fig. S5, the energy monotonically decreases over time, revealing the stability of the scheme. At steady state, the final computed bending and Gaussian energies are $E^*_{\rm B} \approx 1.941$ and $E^*_{\rm G} \approx -1.023$. The same simulation has also been carried out in the $r^* - z^*$ plane, exploiting the axial symmetry, with $\epsilon^* = 2h^* = 1$ and two different time steps, $dt^* = 0.8$ and $dt^* = 0.4$, respectively, still obtaining the same behavior. Convergence has also been observed setting $\epsilon^* = 1.5\,h^* = 1$ and $1/\lambda = 40\sqrt{2}$, with the two spheres at distance $R^*/4$ from each other.

With the same parameters, let's take the dumbbell shape obtained by merging the two spheres in a simulation with the sole bending energy, and let's use it as a new initial condition for the Allen-Cahn dynamics where the Gaussian energy is now included. As shown in Supplementary Fig. S6, the dumbbell shape remains substantially unchanged, showing that the configuration is still a local energy minimum and that there exists an energy barrier that prevents it from dividing into two spheres. It is worth noticing that the computed energies are in excellent agreement with the ones reported in [4]. Bending and Gaussian contributions to the energy of the final configuration are $E^*_{\rm B} \approx 1.625$ and $E^*_{\rm G} \approx -5.095 \cdot 10^{-1}$.

Finally, we test a toroidal topology case. The initial condition is a torus with exact circular cross-section of radius $R^* = 10$ and $v \approx 0.6$. The dynamics lead to a torus with a cross-section that is no more perfectly circular, in excellent agreement with [5], both as regards the shape and the energy. Supplementary Fig. S7 shows the energy evolution both with and without the Gaussian energy term. The two dynamics appear to be very similar, confirming that the Gaussian energy term plays no role as long as no topological transitions occur. These axisymmetric simulations have been carried out in a $[0, 40] \times [0, 40]$ computational domain in the $r^* - z^*$ plane with a grid of $60 \times 60$ nodes, $\epsilon^* = 1.5\,h^* = 1$, $1/\lambda = 20\sqrt{2\pi}$, $M^* = 1$ and $dt^* = 1$. With the Gaussian term included, the final computed bending and Gaussian energies are $E^*_{\rm B} \approx 1.831$ and $E^*_{\rm G} \approx -4.813 \cdot 10^{-2}$, respectively. Noteworthy, the computed Gaussian energy is greater than that reported in Table 1, since higher order corrections to the tanh -solution are present, see Section Methods, equation (15). Performing the same simulation with $1/\lambda = 40\sqrt{2\pi}$, $\epsilon^* = 1.5\,h^* = 1$, and the same $dt^*$, the computed energies at the same final time are $E^*_{\rm B} \approx 1.813$ and $E^*_{\rm G} \approx -1.096 \cdot 10^{-2}$. The error with respect to the data reported in Table 1 decreases, since, by reducing the dimensionless thickness $\lambda$, the higher order terms become less and less important, equation (S.25).

In all these simulations, vesicles area and volume are conserved with the same accuracy of the MEP of the main text, as reported in Supplementary Fig. S8.

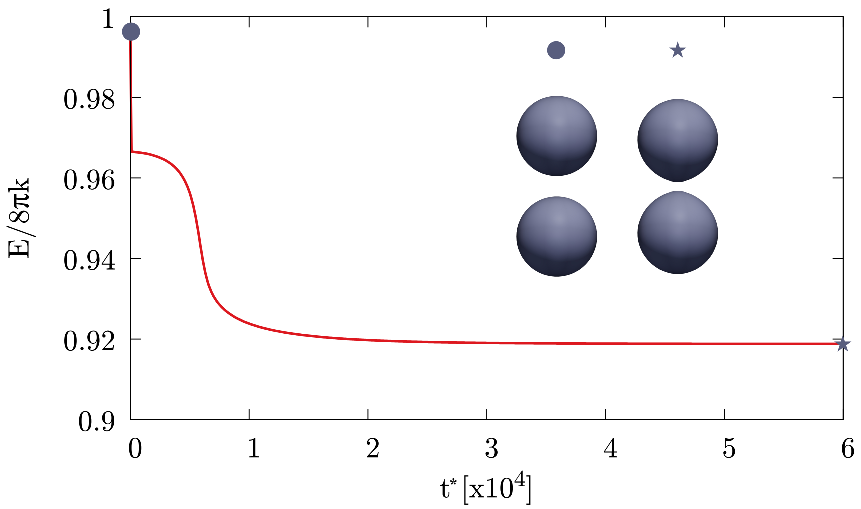


**Supplementary Fig. S5 Relaxation of two close-by spheres.** Two spheres of radius $R^* = 10$ and $R^*/2$ distant from each other do not merge during the Allen-Cahn dynamics in presence of the new phase-field Gaussian energy term. For this simulation we used a $[0, 40] \times [0, 40] \times [0, 66]$ full 3D $x^* - y^* - z^*$ domain with a grid of $40 \times 40 \times 66$ nodes, $\epsilon^* = h^* = 1$, $m^* = 0$, $1/\lambda = 20\sqrt{2}$, $M^* = 1$ and time step $dt^* = 0.8$.

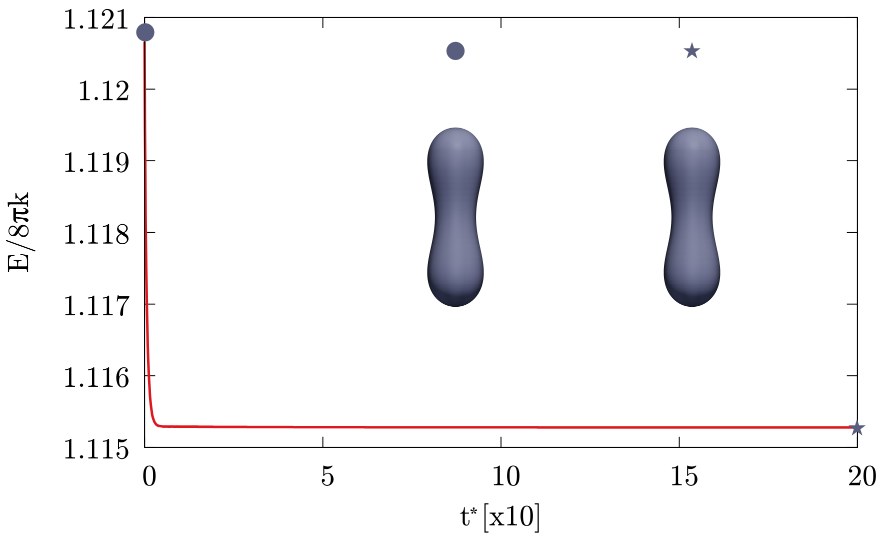


**Supplementary Fig. S6 Relaxation of a prolate.** A dumbbell shape, which is a minimal configuration for the bending energy alone, is also a minimal configuration for the whole Canham-Helfrich energy. This z-axial symmetric simulation has been carried out in a $[0, 28] \times [0, 120]$ computational domain in the $r^* - z^*$ plane with a $42 \times 180$ mesh, $\epsilon^* = 1.5h^*$, $m^* = 0$, $1/\lambda = 40\sqrt{2}$, $M^* = 8$ and $dt = 0.01$.

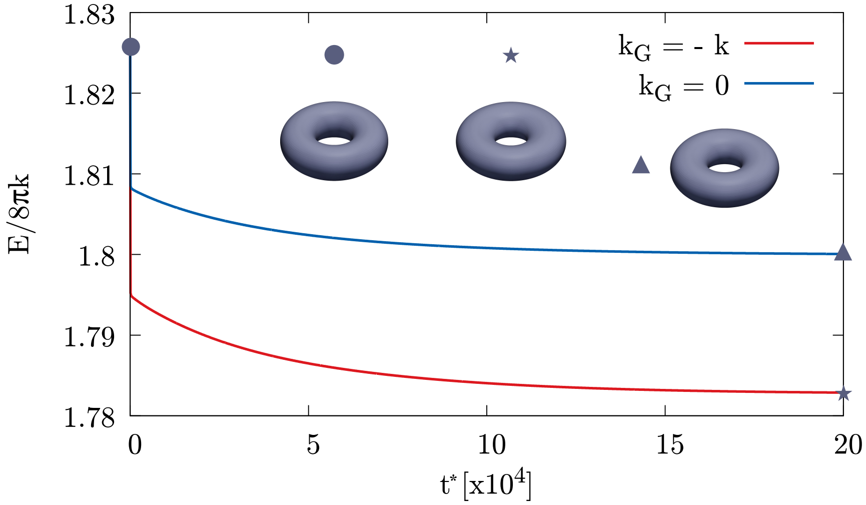


**Supplementary Fig. S7 Relaxation of a torus.** A torus with an exact circular cross-section of radius $R^* = 10$ evolves to a torus with a cross-section that is not perfectly circular. This happens both with $k_G = 0$ (top blue line) and $k_G = -k$ (bottom red line). These z-axial symmetric simulations have been carried out in a $[0, 40] \times [0, 40]$ computational domain in the $r^* - z^*$ plane with a $60 \times 60$ mesh, $\epsilon^* = 1.5h^* = 1$, $m^* = 0$, $1/\lambda = 20\sqrt{2\pi}$, $M^* = 1$ and $dt^* = 1$.

### Area and volume conservation along the MEP

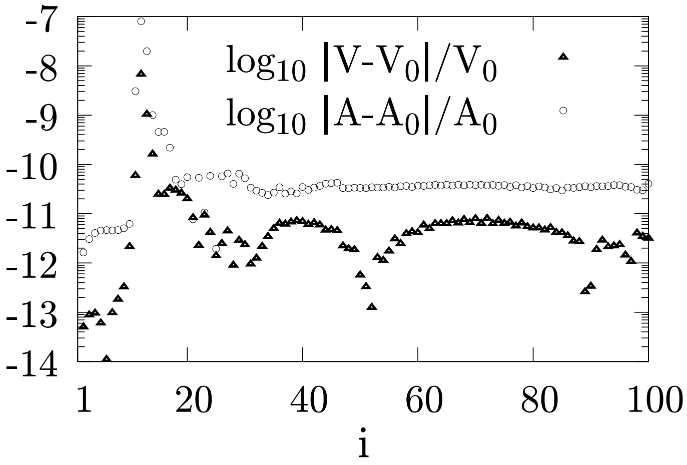


**Supplementary Fig. S8 Errors in preserving area and volume along the MEP.** The effectiveness of the scheme in preserving vesicles area and volume along the MEP of the main text, Fig. 2. Reference values of area and volume are $A_0^* = 1.924392 \cdot 10^5$ and $V_0^* = 5.615982 \cdot 10^6$.

### Functional derivatives

The functional derivative of the energy (S.1) is

$$\frac{\delta E}{\delta \phi} = \frac{\delta E_{\mathrm{B}}}{\delta \phi} + \frac{\delta E_{\mathrm{G}}}{\delta \phi}. \tag{S.29}$$

The bending term is well known in literature and its explicit expression is

$$\frac{\delta E_{\mathrm{B}}}{\delta \phi} = k\, \frac{3}{2\sqrt{2}}\, \epsilon \left[ \nabla^2 \psi_{\mathrm{B}} - \frac{\psi_{\mathrm{B}}}{\epsilon^2} (3\phi^2 - 1 + 2\sqrt{2}\epsilon m \phi) \right]. \tag{S.30}$$

As regards the Gaussian term, noticing that

$$\boldsymbol{\nabla} \cdot \left( \boldsymbol{\nabla}\phi \nabla^2 \phi - \frac{\boldsymbol{\nabla}|\boldsymbol{\nabla}\phi|^2}{2} \right) = (\nabla^2 \phi)^2 + \boldsymbol{\nabla}\phi \cdot \boldsymbol{\nabla}\nabla^2\phi - \frac{\nabla^2 |\boldsymbol{\nabla}\phi|^2}{2}, \tag{S.31}$$

integration by parts of (S.4) leads to

$$E_{\mathrm{G}}[\phi] = k_{\mathrm{G}}\, \frac{35}{8\sqrt{2}}\, \epsilon^3 \int_{\Omega} \tilde{\psi}_{\mathrm{G}}\, dV \ , \tag{S.32}$$

$$\tilde{\psi}_{\mathrm{G}} = \frac{\boldsymbol{\nabla}|\boldsymbol{\nabla}\phi|^2 \cdot \boldsymbol{\nabla}|\boldsymbol{\nabla}\phi|^2}{2} - (\boldsymbol{\nabla}|\boldsymbol{\nabla}\phi|^2 \cdot \boldsymbol{\nabla}\phi)\nabla^2\phi \,. \tag{S.33}$$

This simplifies the computation of the functional derivative, which turns out to be

$$\begin{aligned} \frac{\delta E_{\mathrm{G}}}{\delta \phi} = k_{\mathrm{G}}\, \frac{35}{8\sqrt{2}}\, \epsilon^3 \Big\{ & 2\boldsymbol{\nabla} \cdot [(\nabla^2 |\boldsymbol{\nabla}\phi|^2)\boldsymbol{\nabla}\phi] \\ & + \boldsymbol{\nabla} \cdot (\nabla^2\phi \boldsymbol{\nabla}|\boldsymbol{\nabla}\phi|^2) - \nabla^2(\boldsymbol{\nabla}|\boldsymbol{\nabla}\phi|^2 \cdot \boldsymbol{\nabla}\phi) \\ & - 2\boldsymbol{\nabla} \cdot [\boldsymbol{\nabla}\phi \boldsymbol{\nabla} \cdot (\nabla^2\phi \boldsymbol{\nabla}\phi)] \Big\}. \end{aligned} \tag{S.34}$$

In a more readable form:

$$\frac{\delta E_{\rm G}}{\delta \phi} = k_{\rm G}\,\frac{105}{2\sqrt{2}}\,\epsilon^3 \left[\frac{\partial^2\phi}{\partial x^2}\left(\frac{\partial^2\phi}{\partial y\partial z}\right)^2 + \frac{\partial^2\phi}{\partial y^2}\left(\frac{\partial^2\phi}{\partial x\partial z}\right)^2 + \frac{\partial^2\phi}{\partial z^2}\left(\frac{\partial^2\phi}{\partial x\partial y}\right)^2 - \frac{\partial^2\phi}{\partial x^2}\frac{\partial^2\phi}{\partial y^2}\frac{\partial^2\phi}{\partial z^2} - 2\frac{\partial^2\phi}{\partial x\partial y}\frac{\partial^2\phi}{\partial x\partial z}\frac{\partial^2\phi}{\partial y\partial z}\right], \tag{S.35}$$

which, in the axisymmetric case, further simplifies to

$$\frac{\delta E_{\rm G}}{\delta \phi} = k_{\rm G}\,\frac{105}{2\sqrt{2}}\,\epsilon^3\,\frac{1}{r}\frac{\partial\phi}{\partial r}\left[\left(\frac{\partial^2\phi}{\partial r\partial z}\right)^2 - \frac{\partial^2\phi}{\partial r^2}\frac{\partial^2\phi}{\partial z^2}\right]. \tag{S.36}$$

Furthermore, the functional derivatives of the area (17) and volume (18) are

$$\frac{\delta A}{\delta\phi} = \frac{3}{2\sqrt{2}}\,\epsilon\left[\frac{1}{\epsilon^2}\phi(\phi^2-1) - \nabla^2\phi\right], \tag{S.37}$$

$$\frac{\delta V}{\delta \phi} = \frac{1}{2}. \tag{S.38}$$

In conclusion, the functional derivative of the augmented energy (21) is

$$\frac{\delta \bar{E}}{\delta\phi} = \frac{\delta E_{\rm B}}{\delta\phi} + \frac{\delta E_{\rm G}}{\delta\phi} + \gamma\frac{\delta A}{\delta\phi} + \Delta p\frac{\delta V}{\delta\phi} + + M_1(A[\phi]-A_0)\frac{\delta A}{\delta\phi} + M_2(V[\phi]-V_0)\frac{\delta V}{\delta\phi}\ . \tag{S.39}$$

**Geometrical interpretation of the neck energy**

As discussed in the main text, during the topological transition, a neck, defined as the region where the Gaussian energy density is positive (see Supplementary Fig. S9 sketching a vesicle configuration), forms, acting as a bridge between the two initially separated vesicles. With reference to Fig. 3 of the main text, proceeding to the right along the MEP, beyond the saddle point, the computed Gaussian energy of the neck progressively decreases. In order to get a geometrical understanding of this decrease, for each image along the MEP, we extract the $\phi = 0$ isoline in the $r - z$ plane from the phase-field and find its interpolating polynomial $r = r(z)$. By resorting to the Gauss-Bonnet theorem,

$$\int_\Gamma G\; dS = 2\pi\chi(\Gamma) - \int_{\partial\Gamma} k_{\rm g}\; dl, \tag{S.40}$$

the total curvature of the neck (a surface with Euler characteristic $\chi = 0$) is (minus) the integral of the geodesic curvature $k_{\rm g}$ along the neck boundary (made up of the two circles of radius $r(\pm Z)$ shown in yellow in Supplementary Fig. S9). The square of the geodesic curvature of these circles is $k_{\rm g}^2 = k^2 - k_{\rm n}^2$, where $k = 1/r(\pm Z)$ and $k_{\rm n}$, namely the normal curvature, is the curvature of the vesicle section with the plane containing both the surface normal and the tangent to the circles, evaluated at the neck boundary. The sketch of Supplementary Fig. S9 shows in red the osculating circle defined by the radius of normal curvature

$$\frac{1}{k_{\rm n}} = r(\pm Z)\sqrt{1 + \left.\frac{dr}{dz}\right|^2_{\pm Z}}\ . \tag{S.41}$$

The sharp interface Gaussian energy of the neck is

$$\frac{E^{\rm neck}_{\rm G,CH}(Z)}{8\pi k} = \frac{r(\pm Z)k_{\rm g}}{2} = \frac{\sqrt{1-(r/R_{\rm n})^2}}{2}\ , \tag{S.42}$$

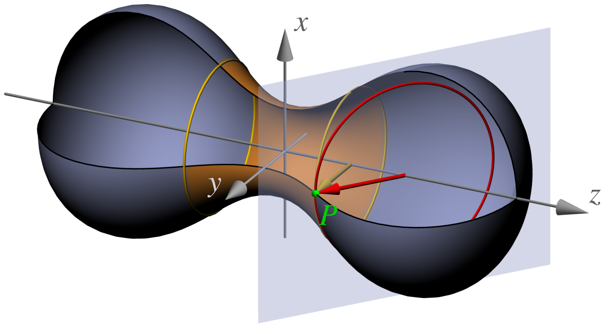


**Supplementary Fig. S9 Sketch of the neck geometry.** The neck region, highlighted in orange, is delimited by the two yellow circles (the curvature radius $r(Z)$ is depicted as a yellow arrow). The red circle is the osculating circle to the vesicle cross-section with the cutting (shaded) plane passing through the neck boundary and containing both the surface normal and the tangent to the yellow circle at the point P highlighted in green. The radius $R_{\rm n}$ of the osculating circle is shown as a red arrow.

where $R_{\rm n} = 1/k_{\rm n}$. From the insets of Fig. 3, near the saddle point (configuration denoted by the triangle) $R_{\rm n} >> r(\pm Z)$, leading to $k_{\rm g} \approx 1/r(\pm Z)$. Moving away from the saddle point, the two curvature radii become eventually comparable, $R_{\rm n} \approx r(\pm Z)$, leading to $k_{\rm g} \approx 0$. The main plot in the figure provides the actual Gaussian energy of the neck as computed from the phase-field (blue line) compared to the sharp-interface approximation (orange line with dots). The agreement is excellent as long as different membrane segments do not approach each other to a distance smaller than the bilayer thickness. Indeed, the agreement progressively deteriorates when getting closer to the saddle point, due to the increasing curvature of the membrane generatrix. Actually, one could better and better reproduce the sharp interface energy also in this stage by reducing the regularizing parameter $\lambda$. On the other hand, from a physical point of view, the thickness of the bilayer is finite, making the sharp interface model inappropriate when the saddle point is approached. Overall, these results confirm the accuracy of the proposed phase-field expression for the Gaussian curvature.

## SUPPLEMENTARY REFERENCES

[1] Jan Steinkühler, Roland L. Knorr, Ziliang Zhao, Tripta Bhatia, Solveig Mareike Bartelt, Seraphine Valeska Wegner, Rumiana Dimova, and Reinhard Lipowsky, "Controlled division of cell-sized vesicles by low densities of membrane-bound proteins," Nature Communications **11** (2020).

[2] Xiaoqiang Wang, "Asymptotic analysis of phase field formulations of bending elasticity models," SIAM J. Math. Anal. **39**, 1367–1401 (2008).

[3] Selim Esedoglu, Andreas Ratz, and Matthias Roger, "Colliding interfaces in old and new diffuse-interface approximations of willmore-flow," Communications in Mathematical Sciences **12**, 125–147 (2012).

[4] Seifert, Berndl, and Lipowsky, "Shape transformations of vesicles: Phase diagram for spontaneous- curvature and bilayer-coupling models." Physical review. A, Atomic, molecular, and optical physics **44 2**, 1182–1202 (1991).

[5] Seifert, "Vesicles of toroidal topology." Physical review letters **66 18**, 2404–2407 (1991).
